# Communication experiences of deaf and hearing-impaired competitive swimmers and their coaches in South Africa

**DOI:** 10.4102/sajcd.v73i1.1142

**Published:** 2026-04-22

**Authors:** Dhanashree Pillay, Caitlin Lewington

**Affiliations:** 1 Department of Audiology, Faculty of Humanities, University of the Witwatersrand, Johannesburg, South Africa

**Keywords:** athlete, deaf, Sign Language, inclusivity, coaches, swimming

## Abstract

**Background:**

Effective communication between deaf swimmers and their coaches is vital for the athlete. Swimmers who wear amplification devices are often disadvantaged when engaging in the sport, as the devices cannot be worn, thereby impacting communication.

**Objectives:**

To explore the communication experiences between deaf and hearing-impaired (D and HI) competitive swimmers and their coaches.

**Method:**

An exploratory phenomenological qualitative research design using online questionnaires was utilised. Non-probability, purposive sampling was employed to select 11 participants (eight D and HI competitive swimmers and three coaches).

**Results:**

Facilitators of effective communication included lip reading, the use of specific made-up signs and writing. Made-up signs are utilised; however, Sign Language was not considered to be the main form of communication because of the lack of knowledge by coaches, thus making communication difficult for swimmers who use Sign Language as their first language. Not being able to wear amplification devices when swimming and the lack of visual-based alerting systems in the swimming arena were identified as barriers. Communication between D and HI swimmers and a deaf coach was described as ‘seamless’, as an understanding of communication modes is established while working together. Inclusivity in the swimming environment is evident; however, there are instances where D and HI swimmers feel isolated.

**Conclusion:**

Communication experiences within the swimming environment included challenges and barriers for both groups of participants. The coach’s relationship with the D and HI swimmer plays a major role in the effectiveness of communication.

**Contribution:**

Fostering positive relationships between coaches and D and HI swimmers is mutually beneficial. Audiologists can play a role in encouraging and facilitating communication strategies with such sports men and women.

## Introduction

Communication is integral to the functioning of any society, and without effective communication, members of society are often excluded from everyday activities. Communication is a vital form of social participation, and the Deaf and hearing-impaired (D and HI) community may be disadvantaged if communication is not inclusive. Communication strategies are necessary for effective communication when hearing and D and HI individuals interact, especially during social activities. Various sectors, including economic, educational, social and sporting domains, rely on effective communication to function adequately. Communication in sporting environments can have an overall impact on the performance and inclusivity of athletes. Ensuring that all athletes, regardless of ability, have equal opportunities to participate in a communicatively accessible environment is essential for fostering inclusivity and equity in both training and competitive settings (Houinato, 2017). Hearing loss can be either congenital or acquired as a result of factors such as ageing, disease, noise exposure, genetics or trauma. Individuals classified as D and HI have a compromised auditory system, which can significantly impact their ability to engage in verbal communication (Palmer, 2018).

Deaf and hearing-impaired athletes who wear hearing aids or cochlear implants are often put at a disadvantage when engaging in sports, as sweat, water or moisture can cause damage to the amplification devices, and the athletes are therefore unable to use these devices during sporting activities (Best et al., 2002). Consequently, these athletes often remove the assistive devices when participating, which hinders communication, especially in the context of swimming. While D and HI athletes do not require physical modifications to participate in sports, they may encounter challenges related to communication accessibility (Houinato, 2017). Communication is necessary to assist with the inclusion of D and HI individuals in a swimming environment where most of the swimmers are hearing. Targeted support for the D and HI population, as well as awareness of how to communicate with individuals who are either deaf or have a hearing impairment, is necessary (Kurková & Scheetz, 2016). Deaf awareness allows for the social inclusion and integration of athletes who are D and HI into the hearing community. Communication differences between hearing athletes and D and HI athletes require individuals to actively work on inclusivity and the integration of the two groups with different communication modes. Communication strategies between these individuals help in acknowledging differences (Kurková & Scheetz, 2016). The acknowledgement and acceptance of different communication modes between D and HI athletes and hearing athletes will allow for the assimilation of the two groups, as their differences are embraced, which will allow D and HI athletes to engage more with the hearing population (Tanure Alves et al., 2021). Competitive swimmers rely on coaching support to enhance their skills (Hammond et al., 2019), and effective communication between D and HI swimmers and their coaches is vital.

Coaches working with athletes who have disabilities often face challenges related to communication, as they may not possess the necessary skills to effectively interact with athletes with disabilities (Hammond et al., 2019). To foster inclusive sporting environments, specific adaptations should be made or introduced to overcome barriers that arise when an athlete has a disability (Hammond et al., 2019). The coaches therefore need to be trained on different communication strategies that can assist in effective communication. The communication obstacles faced by coaches often make inclusivity and participation difficult for athletes who are D and HI, as they are unable to be as involved in the communication mode of their hearing counterparts. Competitive sporting events, including the Olympics and Paralympics, should prioritise equal access to communication resources to ensure that all athletes can fully participate and compete equally.

When focusing on deaf sport in South Africa, the South African Deaf Sports Federation (SADSF) serves as the national governing body responsible for the administration and coordination of various sporting codes for D and HI athletes. The SADSF oversees all areas of deaf sport in South Africa, with a focus on identifying and nurturing sporting talent within all provinces. The SADSF has been responsible for campaigning for better facilities as well as for the provision of training for these athletes to move towards a more inclusive sporting environment. Despite the advancements in inclusivity being made by the governing bodies within South Africa, ongoing efforts are necessary to address communication barriers specifically to ensure that D and HI athletes have equal access to these environments, which will enable them to thrive within their sporting codes.

The Paralympics has enhanced awareness and understanding of disability in sport (Foster et al., 2018). However, there is no specific classification for D and HI athletes unless they have an additional disability, which qualifies them for Paralympic participation (Ercan et al., 2016). This gap led to the creation of the Deaflympics, an international event for deaf athletes, where competitors must have a hearing threshold of 55 dB or worse in their better ear (Ercan et al., 2016). The Deaflympics provides a setting adapted to the communication and cultural needs of deaf athletes, who are otherwise physically able-bodied but may face communication barriers (Ercan et al., 2016). South African swimmer Terrence Parkin exemplifies deaf sporting success, having won 29 Deaflympic gold medals (Scheepers, 2021). Overall, the Paralympics has helped transform negative attitudes towards disability by focusing on achievement rather than impairment (Gold & Gold, 2007).

The study aimed to explore the communication experiences of D and HI competitive swimmers and their coaches in South Africa. By investigating their interactions, the study sought to provide insight into the effectiveness of communication strategies, as well as the challenges and facilitators that shape these experiences.

### Objectives

The research had four key objectives. Firstly, it aimed to identify and explore the different communication strategies utilised between D and HI competitive swimmers and their coaches. Secondly, the study sought to document the barriers that hinder effective communication within the competitive swimming environment. Thirdly, it aimed to highlight the facilitators that enhance communication strategies in this context. Fourthly, the study examined how inclusivity for D and HI swimmers is implemented within the sport.

## Research methods and design

### Research design

The study employed a phenomenological qualitative research design to explore the communication experiences of D and HI competitive swimmers and their coaches. Qualitative research allows for an in-depth understanding of meaning, perspective and experiences, making it particularly suited for capturing the nuanced interactions between swimmers and coaches (Hammarberg et al., 2016).

### Study population and sampling

This study employed a non-probability, purposive sampling strategy to deliberately recruit participants from a specific population, ensuring that the sample consisted of individuals with relevant lived experiences. The community of D and HI competitive swimmers and swimming coaches is sparse in South Africa. Mr X, a hearing-impaired swimming coach with cerebral palsy who has coached both hearing and D and HI swimmers since 2009, assisted in obtaining the participants for the study. He shared the invitation to participate with D and HI swimmers with whom he had been in contact during his coaching career. Mr X has been involved in many aspects of South African swimming and accompanied swimmers to the Deaflympics; however, he stated that many D and HI swimmers had retired after the 2017 Deaflympics, leaving very few in the country.

A total of eight D and HI competitive swimmers were selected through various swimming organisations and private swimming clubs. This approach allowed for the inclusion of participants actively engaged in competitive swimming, thereby providing rich, context-specific insights into their communication experiences with coaches. Additionally, three swimming coaches were recruited for the study. Participants were asked to share information about the study with their coaches, and those who were willing to participate contacted the researchers. The limited number of coaches specialising in training swimmers with disabilities in South Africa influenced the sample size.

### Swimmer participant criteria

#### Inclusion criteria

- Competitive swimmers diagnosed as deaf or hearing-impaired.
- Swimmers who swim competitively and belong to a swimming school or a private swimming club.
- Swimmers living within South Africa.
- All genders included.
- Aged 10 years and older (parental consent and participant assent were obtained if participants were minors).

#### Exclusion criteria

- Individuals who are illiterate as the research required the participant to complete an online questionnaire.
- Non-competitive D and HI swimmers.

### Coach participant criteria

#### Inclusion criteria

- Coaches of D and HI competitive swimmers.
- Coaches within South Africa.
- Coaches affiliated with Swimming South Africa (the national swimming governing body of South Africa).
- All genders included.
- Coaches could be hearing, deaf or hard of hearing.

#### Exclusion criteria

- Coaches of D and HI non-competitive swimmers.
- Individuals who are illiterate, as the research required the participant to complete a questionnaire.

### Data collection procedure and instrumentation

Non-medical ethical clearance was obtained prior to the data collection process beginning. To facilitate participant recruitment, the researcher contacted a D and HI swimming coach and requested that he facilitate communication between potential participants and the researcher.

Data collection was conducted through online questionnaires (one for coaches and one for swimmers), which were distributed via email (email addresses were provided to the researchers via Mr X after he had received permission to share from potential participants). The questionnaires were developed for this study and contained both open- and closed-ended questions. Given the challenges posed by the coronavirus disease 2019 (COVID-19) pandemic and its restrictions, online questionnaires provided a practical and accessible means of gathering data while ensuring participant safety. Prior to completing the questionnaire, participants received an information sheet detailing the purpose, procedure and logistics of the study.

### Data analysis

The data were analysed using a qualitative inductive thematic analysis approach, which allowed for the identification of recurring patterns or themes within the participants’ responses (Braun & Clarke, 2006). Once all questionnaires were received and collated, the data were systematically categorised, enabling key themes to be identified, which reflected the participants’ perspectives and interactions within the competitive swimming environment.

### Ethical considerations

Ethical clearance to conduct this study was obtained from the University of the Witwatersrand Non-Medical Research Committee on 21 March 2021. The ethical approval number is STA_2021_15.

#### Confidentiality and informed consent and assent

Participants were assigned a number, which was reflected in all published reports, to keep their personal information private. Informed consent and assent were important aspects of any research study. Informed consent and assent were provided by participants or their parents and guardians prior to the completion of the questionnaire.

#### Autonomy

Participants were informed that they were not obliged to participate in the study and that they would not receive any remuneration for participating in the study. All participants could freely withdraw from the study at any moment without consequence and were informed prior to the completion of the questionnaire.

#### Trustworthiness

To ensure the trustworthiness of this study, the criteria of credibility, dependability, transferability and confirmability were applied (Lincoln & Guba, 1985). Credibility was established through the method implemented, including the use of purposive sampling to recruit participants with relevant lived experiences and the application of thematic analysis to accurately capture their perspectives. Dependability was maintained by providing a clear and detailed account of the research process. Transferability was addressed by offering descriptive data that contextualises the communication experiences of D and HI competitive swimmers and their coaches, enabling potential applicability to similar competitive sporting contexts. Finally, confirmability was ensured through representing the participants’ experiences as reported in their questionnaires, which enhances the reliability of the study’s findings.

## Results

The results are presented according to the objectives and themes identified. Participants were categorised as:

- Participants one to eight include D and HI competitive swimmers.
- Participants nine to eleven include the coaches of D and HI swimmers.

### Objective 1: To explore the different communication strategies used by D and HI competitive swimmers and their coaches

Participant groups indicated lip reading, the use of a whiteboard and writing as positive strategies used during communication. The main communication strategy noted by 7 out of the 11 total participants was lip reading. Another commonality that was noted by 6 out of the 11 participants was the use of ‘made up signs’ (signs that individuals created based on their personal choices and communication strategies). The two participant groups (the competitive swimmers and the coaches) adopted these communication strategies through natural use of signs or through general trial and error when attempting to form a workable communication system. Participants were asked how they learnt about and adopted communication strategies. The following quotes from participants describe their responses:

‘Speech therapy as a child and my parents ensuring that lip reading, and word pronunciation were built into how they communicated with me. This was my normal.’ (P1, female, 21 years old, hearing aid user and HI)

‘I have always been able to lip read, and my coach and I just formed this understanding of how we communicate. The writing on the white board is also just a quick easy way that is used for the whole team.’ (P3, female, 22 years old, bilateral cochlear implants and HI)

‘Trial and error mostly. I had many coaches in many different sized teams and it went from having imposter syndrome to being able to openly say “hey these are my needs, and this is how you can meet them to ensure I am successful in this sport.”’ (P7, female, 27 years old, hearing aid user and HI)

‘I learnt at a young age to accept deaf swimmers and never treat them any different. And as a coach, in the beginning I did struggle, but I do build a bond with my swimmers that we understand each other I always ask them how they would prefer me to coach them, and what would help them best. If they teach me something, I will use this as well.’ (P9, male, 46 years old, 17 years coaching and no hearing loss)

‘Me being me I strongly agree as I make sure that I have built a relationship with my coach to ensure that we both get out of the relationship what we need. Communication has always been effective but the understanding of being hearing impaired and your special needs are sometimes dismissed.’ (P1, female, 21 years old, hearing aid user and HI)

One of the coaches is deaf; thus communication strategies were an additional learning process, and Sign Language was used, as per the quoted response from the coach:

‘I am Deaf, so, it is so natural. With hard of hearing, I can also use sign English.’ (P11, male, 49 years old, Sign Language, 12 years coaching and deaf)

### Objective 2: To document any barriers to communication strategies within the sport of competitive swimming

Participants were asked: ‘*What were some of the challenges faced within the swimming environment that were related to communication, and to describe instances where they experienced exclusion within the swimming environment due to communication?*’

#### Theme 1: Use and non-use of Sign Language in the swimming environment

The majority of swimmers reported that communication within the swimming environment is not through Sign Language even though they might have a ‘made-up signing system’ with their coaches. The following participant responses illustrate the underutilisation of Sign Language:

‘I do not know how to speak Sign Language and nor do most people including coaches.’ (P4, male, 20 years old, cochlear implants and HI)

‘Minor Sign Language is used in common instances in training such as times, when to go, distances etc. More in technical instances.’ (P7, female, 27 years old, hearing aid user and HI)

‘While I speak and don’t sign, since I can’t hear in the water, my coach and I made up our own signs for easy communication.’ (P8, male, 24 years old, hearing aid user and HI)

‘I know of a few coaches who have similar qualities as myself, with wanting to help these swimmers. I do however find that there are coaches who are not willing to step out of their comfort zones with average swimmers.’ (P9, male, 46 years old, 17 years coaching and no hearing loss)

‘New coaches sometimes get impatient …’ (P10, female, 33 years old, 10 years coaching and no hearing loss)

‘There will be communication barriers or misunderstandings or frustrations … Hearing swimmers don’t know Sign Language. Very few will have [*patience*] to try to communicate. Swimming environment is about competition, hence no time for deaf.’ (P11, male, 49 years old, Sign Language, 12 years coaching and deaf)

#### Theme 2: Challenges faced by deaf and hearing-impaired swimmers and their coaches

Participants were asked to provide details pertaining to the challenges faced in the swimming environment. A visual race starting system is lacking in most swimming environments, which disadvantages D and HI swimmers, as the only indication of the start of the race is an auditory shotgun indicator. Two participants reported having this experience:

‘If the start of a race was a whistle blow, I was in trouble.’ (P1, female, 21 years old, hearing aid user and HI)

‘Another instance is in school for galas there was no light at the time for diving in so I would use the hand gesture and always start about 2 seconds later.’ (P3, female, 22 years old, bilateral cochlear implants and HI)

Another challenge that D and HI swimmers encounter is the inability to use a hearing aid or cochlear implant while swimming, as stated by three swimmers:

‘Open water swimming makes it harder as you have to take out your cochlear implants long before the race and people are talking and you feel very lost or confused and at the end of 1km at midmar, when you have to walk through the crowd to find your family I feel more “alone” as I wouldn’t be able to ask someone else for help shows the anxiety that comes with it.’ (P2, female, 26 years old, hearing aid user and HI)

‘Unfortunately, the pool is one of the few places where you cannot wear hearing aids. So, things like the start, calling your name for an award, calling your race to report, things alike that can be incredibly stressful. Coaches will always work with you, most of the time so the biggest standout issue is the communication with other hearing swimmers.’ (P7, female, 27 years old, hearing aid user and HI)

‘Not being as social with teammates in the pool. Though I do and can talk to them normally out the pool.’ (P8, male, 24 years old, hearing aid user and HI)

Each swimmer brings a challenge for coaches, as a communication system must be tailored to the swimmer individually:

‘I only really find it challenging in the beginning when meeting new deaf swimmers, as we have to learn how to communicate with each other effectively. I try my best to put myself in their shoes, as you need to be willing to try and fail and keep trying to communicate as best you can as a coach in order to make your swimmer feel as normal as ever.’ (P9, male, 46 years old, 17 years coaching and no hearing loss)

### Objective 3: To document any facilitators to communication strategies that exist within competitive swimming

#### Theme 3: Interactions with other D and HI swimmers

The participants’ interactions with other D and HI swimmers vary based on their swimming environment, as it is dominated by hearing swimmers. Two of the swimmers have not had any interaction with other D and HI swimmers, and six of the swimmers have interacted with D and HI and hearing swimmers. For those who have interacted with other D and HI swimmers, it was noted that there were mixed responses, as one participant noted that the experiences with other D and HI swimmers were:

‘Great! We all understand each other. We are part of deaf Community, so we support each other.’ (P2, female, 26 years old, hearing aid user and HI)

In other instances, participants noted that their interactions with other D and HI swimmers were:

‘Often awkward as not all deaf swimmers have a hearing device like me. I often cannot sign well enough to communicate clearly. Thankfully most have good lip reading.’ (P8, male, 24 years old, hearing aid user and HI)

Another participant shared how interactions with other D and HI swimmers allowed a feeling of being understood because of a shared lived experience:

‘Many of them use the same methods as I do. Looking at the workout or plan before, in writing and then using basic gestures and writing during the workout. The in-depth coaching occurs out of the water, or after the swim. I feel many of my likewise peers also experience loneliness and being left out during competitions and meets because teammates don’t always remember we cannot hear.’ (P7, female, 27 years old, hearing aid user and HI)

#### Theme 4: Relationship building and communication with coaches

Participants were asked to describe their communication experiences with their coaches. Participants related positive communication to relationship building. The following participant responses show how effective communication is achieved within the swimming environment when relationships are developed between swimmers and coaches:

‘I’ve built a relationship with my coach to ensure an understanding on how I need to be communicated with.’ (P1, female, 21 years old, hearing aid user and HI)

‘My coaches understand me, they talk slower, and they are willing to help and learn.’ (P2, female, 26 years old, hearing aid user and HI)

‘Communication is relatively good in the swimming environment, and everyone understands where I stand with how I can communicate. If I don’t catch on what my coach says my team mates often just fill me in … It is positive because my coach is attentive to me and always makes sure I understand what to do. We have a relationship as well which I believe is very important.’ (P3, female, 22 years old, bilateral cochlear implants and HI)

‘I think we adapt, and communication eventually becomes beneficial and effective as you age and understand your needs and limitations. In the younger years it is very ineffective but as you age and learn to communicate better things get better … Coaches are typically caring and receptive in nature. Since they are coaches, they understand when you need to coach them back. Because of the nature of their profession communication is very important to them which makes it easy to work with them. As mentioned in the previous question, it is learning one’s own limits and extent of your own disability which poses challenges in this realm.’ (P7, female, 27 years old, hearing aid user and HI)

‘Basic lipreading understanding has helped me communicate in the water with teammates. Out of water I have my cochlear implant on and can communicate as well as any hearing person … Working with coaches that are willing to assist and understand.’ (P8, male, 24 years old, hearing aid user and HI)

Coaches stated that effective communication results when:

‘I make sure to give each group the communication they might need.’ (P10, female, 33 years old, 10 years coaching and no hearing loss)

‘Deaf swimmers and hearing swimmers communicate with me very well [*which shows that there is a definite understanding and no unresolved miscommunication within these swimming environments*].’ (P11, male, 49 years old, Sign Language, 12 years coaching and deaf)

‘As coach himself is deaf, he is very aware.’ (P5, male, 15 years old, Sign Language and deaf)

‘I think most coaches are aware if explained to properly and can help one on one, but I believe my coach has a lot more experience therefore I can do swimming sessions in a group.’ (P3, female, 22 years old, bilateral cochlear implants and HI)

‘Deaf swimmers should understand session plans as well as the hearing do. They shouldn’t be treated any differently, however, they do need the extra attention when it comes to explaining sessions, drills, or technique changes.’ (P9, male, 46 years old, 17 years coaching and no hearing loss)

### Objective 4: To explore whether and how inclusivity of D and HI swimmers is employed within the sport

#### Theme 5: Exclusion in the swimming environment

Participants were required to describe experiences where they felt excluded in the swimming environment. It was reported that most swimmers have not experienced a time when they were purposefully excluded from any situation within the swimming environment; however, some participants reported that they have experienced instances where they were excluded, as they felt isolated because of a barrier to communication within the swimming pool. This can be seen in the following participants’ responses:

‘Not been able to fully understand set changes in the water that were communicated made me exposed and vulnerable. Also, you constantly feel like people are talking about you and you don’t know what they are saying so you naturally start withdrawing from social interactions as you need to be communicated with differently so that you can lip read or you get the wrong message … It was tough and if I could avoid the interrogation of what made me different I did so if no one spoke to me I was okay with it. This does not mean I was happy, but I accepted that some people are just uneducated when it comes to persons with disabilities.’ (P1, female, 21 years old, hearing aid user and HI)

‘I think the only time I feel excluded is when we are resting in between sets and people are chatting around me but I am not part of that conversation. Luckily the rests are very quick so it’s not too detrimental to my whole swimming experience.’ (P3, female, 22 years old, bilateral cochlear implants and HI)

‘There were many instances during training where I was left out of natural conversations during practices which led me to feel left out. I was unable to bond with my teammates the same way someone with hearing does. There are also many “in the moment” coaching situations where I couldn’t receive feedback on my swim … Communication is normal out of the pool but within the pool I shy away from conversation in fear that I do not understand or in fear that the person won’t have the patience to repeat themselves.’ (P7, female, 27 years old, hearing aid user and HI)

‘I would imagine myself talking to them more if I could hear in the water. I often avoid communicating to avoid awkward misunderstanding.’ (P8, male, 24 years old, hearing aid user and HI)

#### Theme 6: Inclusion in the swimming environment

There were participants in this study who experienced instances of exclusion and inclusion, and their quotes fall under both themes. On the contrary, some participants feel that communication is not a factor that results in their exclusion in the swimming environment; they experienced team collaboration where other teammates or officials aided in their inclusion:

‘Excellent. They are all very welcoming and help me if I don’t understand.’ (P2, female, 26 years old, hearing aid user and HI)

‘They are very supportive and understanding of my impairment and try their best to still include me and chat to me. They don’t treat me any less than the next teammate.’ (P3, female, 22 years old, bilateral cochlear implants and HI)

‘Communication would not be a reason for me to feel or to be excluded from … Perfectly normal, no negatives.’ (P4, male, 20 years old, cochlear implants and HI)

‘Kabelo hearing help me and Amkele. (Hearing teammate helps the deaf swimmers) … Hearing swimmers help us.’ (P5, male, 15 years old, Sign Language and deaf)

‘Never been excluded thanks to decent coaches and race officials.’ (P8, male, 24 years old, hearing aid user and HI)

‘My philosophy is to help everyone who trains in my squad. I will go the extra mile to help swimmers who share the same passion as I do. Everyone should get treated equally and fairly … My team are very accommodating and often try communicate the same way I do. They have learned from watching my hands on pool deck, and often will try help where they can. We look after each other as one swimming family.’ (P9, male, 46 years old, 17 years coaching and no hearing loss)

## Discussion

The major barrier for D and HI athletes in accessing a sporting environment with other hearing athletes or coaches is accessible communication (Foster et al., 2018). Integration of D and HI individuals into sport communities is restricted by the lack of awareness and knowledge within the hearing population (Foster et al., 2018).

Lip reading is the leading communication strategy identified; this is concerning, as only 30% of English is readable through lip reading, which results in 70% of information being an educated guess (Abou-Abdallah & Lamyman, 2021). According to Daisy (2018), deaf individuals choose to assimilate and to fit in with the hearing population, as they choose their identity based on what is more beneficial for their swimming performance. The coaches’ bond and understanding of the needs of D and HI swimmers are vital, as this bond can improve communication within the swimming environment. Not all coaches have an understanding and awareness of behaviour and often follow a traditional way of coaching, which fails to support the needs of athletes with special needs (Harvey et al., 2013).

If Sign Language is not considered an accessible communication mode, barriers to communication can be experienced by D and HI swimmers. Sign Language is not well understood by a large portion of society, which makes using Sign Language in general everyday activities difficult, as not many people make use of it, which results in the loss of communication for D and HI swimmers in some instances (Kholis et al., 2019). deaf athletes might be self-conscious since they use nonverbal communication, which is not familiar to the hearing population (Uchida et al., 2015). According to Hammond et al. (2019), coaches have stated that it was the athlete’s responsibility to adapt to mainstream coaching if they chose to participate in sport. When looking at the responses and experiences of D and HI swimmers and their coaches in terms of effective communication from the coaches, this was not the case for the current study, as coaches ensured that all swimmers, including D and HI swimmers, were given the necessary attention and communication.

Social anxiety and the fear of not being understood are common (Karademir, 2015), and this is evident when swimmers cannot use their cochlear implants or hearing aids. Waterproof amplification devices have not been designed to date and should be considered for future development. Other individuals in the swimming environment should be more aware that swimmers may have more difficulties than usual without amplification devices. It is always important to consider visual sensory cues when working with D and HI individuals, as this is a good way of getting their attention (Houinato, 2017). Therefore, a visual light system should be used at all swimming events where D and HI athletes are partaking, as this should not be a challenge that D and HI swimmers face (Houinato, 2017).

To ensure that exclusion does not occur because of poor communication, coaches have to understand each D and HI swimmer’s communication needs, as there is not only one mode of communication that works for all (Stauder, 2019). According to McMaster et al. (2012), it was noted that coaches said the best learning experience for them was the different interactions they had with athletes with disabilities, as they learnt how to cope through their personal experiences. Sport has the powerful ability to foster inclusion and create a sense of identity for all athletes, regardless of their different abilities, as it allows them to be involved in activities from which they are often excluded because of communication difficulties (Kassing et al., 2004). When looking at a sporting environment that is inclusive, the communication needed for each individual or team is not a uniform one-size-fits-all approach (Stauder, 2019). Therefore, emphasis is needed on communication and the different ways of supporting athletes, so that the sporting environment is inclusive for all.

An athlete-coach relationship is important in ensuring the success of an athlete as well as the development and growth of the athlete (Dominteanu, 2011). When a coaching relationship has formed sufficiently, communication will be improved resulting in an overall positive experience for both the athlete and coach. Coaches must be willing to grow and develop together for the athlete-coach relationship to be effective (Dominteanu, 2011). Participation in sport is linked to enjoyment and the development of social support networks (Allender et al., 2006). Team collaboration and integration are important for the inclusion of D and HI athletes. It can also be seen that the specific communication mode made use of by D and HI athletes is based on the degree of hearing loss, and whether the individual makes use of any amplification devices can also have an impact on their inclusion (Kurková & Scheetz, 2016). When the team provides support for communication and team collaboration is achieved, any form of social exclusion of D and HI individuals is alleviated (Kurková & Scheetz, 2016).

## Conclusion

In summary, the research had four key objectives that were met. Deaf and hearing-impaired competitive swimmers and their coaches used different communication strategies within the swimming environment, providing a positive communication experience for both swimmers and coaches. Using different communication strategies such as lip reading, the use of a whiteboard, writing or having a good understanding relationship with the coach can lead to effective communication. Communication barriers were encountered; however, D and HI competitive swimmers as well as their coaches found ways of working around these different aspects based on the good relationships that were forged. Deaf and hearing-impaired competitive swimmers and their coaches faced challenges such as not being able to use amplification devices in the water, as well as instances where no visual cues were given at the start of a swimming race.

The majority of the swimmers have good interactions with other D and HI swimmers, as well as with hearing swimmers, and form team collaborations. It can be concluded that inclusivity in the swimming environment in South Africa is evident; however, there are still instances where D and HI swimmers feel isolated and unable to communicate with other swimmers within their team. Overall, communication within the swimming environment was reported to be positive, highlighting that an effort was being made to try and achieve effective communication and inclusivity within the swimming environment in South Africa.

This study had limitations, which included a small sample size; however, the communication experiences of D and HI competitive swimmers and their coaches were explored, which provides insight into this niche community. The results of the study do not reflect the communication experiences of all D and HI competitive swimmers and their coaches, as the study was restricted to South African participants only. Coronavirus disease 2019 had a significant impact on the data collection process, as the study would have benefited from having one-on-one interviews as the data collection method, as this would have enabled the researcher to probe further into the answers from swimmers and their coaches.

Despite the shortcomings of the study, it contributes to the growing knowledge of communication within South African society, specifically among athletes such as swimmers and their coaches. Valuable information was gained from this study, such as the need for further advocacy for D and HI individuals to create awareness of different ways of communication, thus eliminating the exclusion of D and HI athletes because of a lack of awareness of how to communicate effectively. There is a gap in the research regarding D and HI athletes, especially swimmers, in the South African context. The findings of this study shed light on instances where insufficient communication strategies were being implemented to achieve effective communication. Thus, this guides athletes and coaches towards achieving effective communication. It is recommended that this study be extended to include international D and HI competitive swimmers and their coaches. This provides the opportunity to gain more information regarding the communication experiences of other D and HI competitive swimmers and their coaches.

The final take-home message from this study is: Communication experiences of D and HI competitive swimmers and their coaches in South Africa may vary; however, a positive relationship between both groups of individuals can aid in achieving effective communication.
